# Impaired B cell immunity in acute myeloid leukemia patients after chemotherapy

**DOI:** 10.1186/s12967-017-1252-2

**Published:** 2017-07-10

**Authors:** Meghali Goswami, Gabrielle Prince, Angelique Biancotto, Susan Moir, Lela Kardava, Brian H. Santich, Foo Cheung, Yuri Kotliarov, Jinguo Chen, Rongye Shi, Huizhi Zhou, Hana Golding, Jody Manischewitz, Lisa King, Lauren M. Kunz, Kimberly Noonan, Ivan M. Borrello, B. Douglas Smith, Christopher S. Hourigan

**Affiliations:** 1 Myeloid Malignancies Section, Hematology Branch, National Heart, Lung and Blood Institute, National Institutes of Health, 10 Center Drive Room 10CRC 5-5216, Bethesda, MD 20814-1476 USA; 2 Johns Hopkins University, Baltimore, MD USA; 3 Center for Human Immunology, Autoimmunity and Inflammation, National Institutes of Health, Bethesda, MD USA; 4 National Institute of Allergy and Infectious Diseases, National Institutes of Health, Bethesda, MD USA; 5 Division of Viral Products, Center for Biologics Evaluation and Research, Food and Drug Administration, Silver Spring, MD USA; 6 Office of Biostatistics Research, National Heart, Lung, and Blood Institute, National Institutes of Health, Bethesda, MD USA

**Keywords:** Adaptive immunity, Leukemia, B-cells, T-cells, Influenza vaccination, Immunotherapy

## Abstract

**Background:**

Changes in adaptive immune cells after chemotherapy in adult acute myeloid leukemia (AML) may have implications for the success of immunotherapy. This study was designed to determine the functional capacity of the immune system in adult patients with AML who have completed chemotherapy and are potential candidates for immunotherapy.

**Methods:**

We used the response to seasonal influenza vaccination as a surrogate for the robustness of the immune system in 10 AML patients in a complete remission post-chemotherapy and performed genetic, phenotypic, and functional characterization of adaptive immune cell subsets.

**Results:**

Only 2 patients generated protective titers in response to vaccination, and a majority of patients had abnormal frequencies of transitional and memory B-cells. B-cell receptor sequencing showed a B-cell repertoire with little evidence of somatic hypermutation in most patients. Conversely, frequencies of T-cell populations were similar to those seen in healthy controls, and cytotoxic T-cells demonstrated antigen-specific activity after vaccination. Effector T-cells had increased PD-1 expression in AML patients least removed from chemotherapy.

**Conclusion:**

Our results suggest that while some aspects of cellular immunity recover quickly, humoral immunity is incompletely reconstituted in the year following intensive cytotoxic chemotherapy for AML. The observed B-cell abnormalities may explain the poor response to vaccination often seen in AML patients after chemotherapy. Furthermore, the uncoupled recovery of B-cell and T-cell immunity and increased PD-1 expression shortly after chemotherapy might have implications for the success of several modalities of immunotherapy.

**Electronic supplementary material:**

The online version of this article (doi:10.1186/s12967-017-1252-2) contains supplementary material, which is available to authorized users.

## Background

Acute myeloid leukemias (AML) encompass a heterogeneous group of oligoclonal hematological malignancies characterized by rapid, uncontrolled proliferation of leukemic blasts in the bone marrow and peripheral blood [1]. Initial treatment for AML typically includes intensive cytotoxic chemotherapy intended to achieve a morphological complete remission (CR) in the bone marrow, but current standard of care regimens have poor clinical outcomes, as over half of AML patients will relapse and only a quarter will survive past 5 years [2].

Allogeneic hematopoietic stem cell transplantation (allo-HSCT) is a consolidative immunotherapy strategy to prevent relapse but is itself associated with significant morbidity and mortality [3]. Nevertheless, given the poor clinical outcomes with chemotherapy alone, the addition of other immunotherapeutic modalities to prevent relapse after completion of cytotoxic chemotherapy is an appealing prospect [4–6]. Cancer vaccination against tumor-specific antigens is a logical immunotherapeutic strategy to prevent relapse by further priming an immune response, and induction of leukemia-associated antigen-specific CD8+ T-cells have been observed in response to peptide vaccination, though responses are often short-lived [7–10]. Vaccination to retain remission in AML patients has produced conflicting results, with some groups reporting long-term efficacy and sustained immune response, while others report deletion of high-avidity CD8+ T-cell clones and unsustainable responses [11–14].

Though immunotherapeutic strategies to maintain remission after patients receive intensive chemotherapy are logical and being tested in clinical trials [6], there is an incomplete description of the state of the adaptive immune system in AML patients who have completed chemotherapy. Previous work on peripheral blood lymphocyte recovery in AML after induction demonstrated a skewing of the T-cell compartment towards peripherally expanded oligoclonal activated T-regulatory cells (T-regs) in the time immediately following chemotherapy [15]. Yet, the functional capacity of the immune system in AML patients after the completion of intensive chemotherapy is largely unknown, and this has important implications for the success of any subsequent immunotherapy intended to prevent relapse, especially cancer vaccination and immune checkpoint blockade meant to augment endogenous cellular-mediated anti-leukemic responses.

The intent of the present study was to quantify phenotypic and functional immune abnormalities in a cohort of AML patients that would be considered ideal candidates for immunotherapies intended to prevent relapse. To eliminate the potential confounding effects of testing novel leukemia vaccines of unknown antigenic potency, we instead studied the well-characterized seasonal influenza vaccine with well-known efficacy profiles in healthy subjects. Recent studies have illuminated the presence of influenza vaccine-induced cell signatures that are predictive of robust immune responses across a spectrum of healthy individuals [16, 17]. Influenza is a significant cause of morbidity and mortality in patients with hematological malignancies [18, 19]. Because perturbations in the adaptive immune system after chemotherapy have severe implications for the success of any immunotherapy, especially vaccine-based modalities, we sought to interrogate the recovery and presence or absence of immune cell populations potentially predictive of response to vaccination in AML patients. We performed comprehensive phenotypic, genetic, and functional immune profiling immediately before and 30 days after the seasonal influenza vaccination in ten AML patients in a first remission after consolidation chemotherapy.

## Methods

### Study design

This study was approved by Johns Hopkins University Institutional Review Board (IRB) and conducted in accordance with the Declaration of Helsinki. All patients signed written consent. Patients were eligible for this study if they had completed treatment for AML at least 3 and no more than 156 weeks prior to enrollment, were judged to be in a complete remission, had not yet received the 2012–2013 seasonal influenza vaccine but were due to receive it as standard of care, and had no prior adverse reactions to vaccinations. Patients received the 2012–2013 trivalent inactivated seasonal influenza vaccine. Peripheral blood was collected on the day of vaccination (prior to vaccination) and 30 days post-vaccination. Ten age- and sex-matched healthy donors (HD) were recruited on an IRB approved healthy donor protocol and provided peripheral blood samples.

### Sample collection and processing

Peripheral blood mononuclear cells (PBMCs) were purified by Ficoll-Hypaque density centrifugation from whole blood delivered into Leucosep tubes (Greiner Bio-One, Radnor, PA, USA). Cells were viably cryopreserved in a controlled rate freezer and distributed for RNA isolation, flow cytometry, and ELISPOT assays. Serum from whole blood was also separated using SST tubes, cryopreserved, and aliquoted for microneutralization assays. Whole blood was also drawn into PAXgene Blood RNA tubes (PreAnalytiX, Feldbachstrasse, Switzerland) as an additional source of nucleic acids. Detailed standard operating procedures are included in Additional file 1.

### Microneutralization assay

Viral-neutralizing activity in AML patients who received the influenza vaccination was analyzed via microneutralization assay with MDCK cells based on the methods developed by the pandemic influenza reference laboratories of the Center for Disease Control and Prevention [20]. Neutralizing antibody titers were measured against seasonal influenza type A H3N2, H1N1, and type B vaccine strains. Human sera from patients at baseline and day 30 were tested at a starting dilution of 1:20, followed by serial twofold dilutions. Those that were negative for antibody titers were assigned a titer of <20. All sera were tested in triplicate, and the geometric mean was used in analysis. A fourfold or greater rise in neutralizing antibody titer at day 30 over baseline was considered a positive response (seroconversion).

### Flow cytometry

High-dimensional flow cytometry for comprehensive leukocyte immunophenotyping was performed. Cell suspensions (from viably frozen cell suspensions) were stained as previously described [21, 22], and the staining panel is detailed in Additional file 2: Table S1. Acquisition was performed using a Becton–Dickinson LSRFortessa (BD, San Jose, CA, USA) equipped with five lasers (355, 407, 488, 532, and 633 nm wavelengths) with 22 PMT detectors, optimized as described by Perfetto et al. [23]. Data were acquired using DIVA 8 software (BD) and we recorded a minimum of 50,000 CD4+ T-cells to be able to accurately assess minor cell populations. Post-acquisition analysis was performed using Flowjo version 9.6.2 (Treestar Inc., San Carlos, CA, USA). For analysis, debris and doublets were excluded using light scatter measurements; subsequently, viability stain was used to ensure that only viable cell subsets were included. All major cell populations were identified based on the co-expression of CD45 and a pan lineage marker as described [17, 22]. Each cell population was represented as percentages of the parent population.

Cell-surface PD-1, TIM3, CTLA4, and 4-1BB expression on CD8+ T-cell subsets was also assessed on the AML patients at baseline and HD. Detailed methods can be found in Additional file 1.

### T-cell and B-cell ELISPOT

IFNγ T-cell ELISPOT assays were used to assess CD8+ T-cell function by quantifying the number of cytokine-secreting T-cells (CSCs), and IgG and IgA B-cell ELISPOT assays were used to assess B-cell functionality by enumerating antibody-secreting B-cells (ASCs). Extended methods can be found in Additional file 1.

### Gene expression analysis using microarray

RNA was amplified from 300 ng of total RNA, and single-strand sense cDNA was reverse transcribed, biotinylated, and hybridized to the GeneChip Human Gene 1.0 ST Arrays (Affymetrix, Santa Clara, CA, USA) after fragmentation. The arrays were washed and stained on a GeneChip Fluidics Station 450; scanning was carried out with the GeneChip Scanner 3000 and image analysis was done with the Affymetrix GeneChip Command Console (AGCC) software. Resulting CEL files were processed with Affymetrix Power Tools for probe set summarization, normalization, and log2-transformation (RMA with sketch quantile normalization). Ingenuity Pathway Analysis software (Ingenuity Systems, Redwood City, CA, USA) and Toppgene Suite were used for functional interpretation of gene expression data.

### Deep sequencing of the B-cell receptor heavy chain

Amplification and sequencing of the B-cell receptor (BCR) immunoglobulin heavy (IGH) chain complementarity-determining region 3 (CDR3) region was performed using the immunoSEQ platform (Adaptive Biotechnologies^©^, Seattle, WA, USA). The somatically rearranged CDR3 region was amplified from 1.5 μg genomic DNA isolated from PAXgene tubes using a two-step, amplification bias-controlled multiplex PCR approach as described elsewhere [24, 25], including the Additional file 1.

### Statistical analyses

Two-tailed Mann–Whitney U tests were used to compare variables in 2 sample groups, and F-tests were used to compare data distribution between 2 sample groups. Kruskal–Wallis analysis of variance (ANOVA) tests were used to test more than 2 sample groups simultaneously. All analyses were performed using Prism software (GraphPad, La Jolla, CA, USA), Microsoft Excel, and R/Conductor. Standard deviation of the mean (SEM) values were calculated to summarize frequencies of immune subsets in HD. For all comparisons, p < 0.05 was considered statistically significant, and multiple testing corrections were performed where appropriate.

## Results

### Patient demographics

Demographics and clinical information at baseline of the ten AML patients are summarized in Table 1. The average age of enrolled patients was 53 years (range 28–69) with a median age of 57 years. All patients were in a first complete remission (CR1) following consolidation chemotherapy, and the average time elapsed from the last chemotherapy treatment was 37 weeks (range 4–148 weeks). Complete blood count details are detailed in Additional file 2: Table S2.

**Table 1:** Patient clinical characteristics

Patient	Age at baseline (years)/sex	Diagnosis	Cytogenetics	Induction	Consolidation	Remission status	Weeks since end of chemotherapy	Baseline ALC (/cu mm)
AML 01	67/F	M5	trisomy 8, inv(16)	AcIVP16	HiDAc × 1	CR1	49	1660
AML 02	69/F	MDS to AML	46 XX	7 + 3	Moderate dose Ac	CR1	10	750
AML 03	52/M	N/A	46 XY, NPM1+	FLAM	FLAM	CR1	45	2530
AML 04	48/F	N/A	inv(16)	AcDVP16	HiDAc × 4	CR1	19	810
AML 05	28/F	N/A	46 XX	AcDVP16	AcDAc	CR1	26	1860
AML 06	58/M	N/A	t(8;21)	AcDVP16	HiDAc × 1, AcDAc	CR1	148	2710
AML 07	55/M	M5	46 XY, FLT3-D835+, NPM1+	AcDVP16	HiDAc × 4	CR1	31	1160
AML 08	66/M	M5	46 XY, NPM1+	AcDVP16	HiDAc × 4	CR1	8	650
AML 09	63/M	M4	46 XY, NPM1+	FLAM	FLAM	CR1	34	730
AML 10	28/M	M3 (APL)	t(15;17), FLT3-ITD+	ATRA/D	AcDArsenic	CR1	4	1440

AML subtype under diagnosis defined by the French–American–British (FAB) classification of AML. *MDS* myelodysplastic syndrome, *APL* acute promyelocytic leukemia, *ITD* internal tandem duplication, *NPM1* nucleophosmin, *FLT3* fms-like tyrosine kinase, *ITD* internal tandem duplication, *CR1* first complete remission, *ALC* absolute lymphocyte count. *A/Ac* cytarabine, *I* idarubicin, *VP16* etoposide, *FL* flavoperidol, *M* mitoxantrone, *D* daunorubicin, *ATRA* all trans retinoic acid, *HiD* high dose

### Poor responses of AML patients after chemotherapy to influenza vaccination

**Fig. 1:**
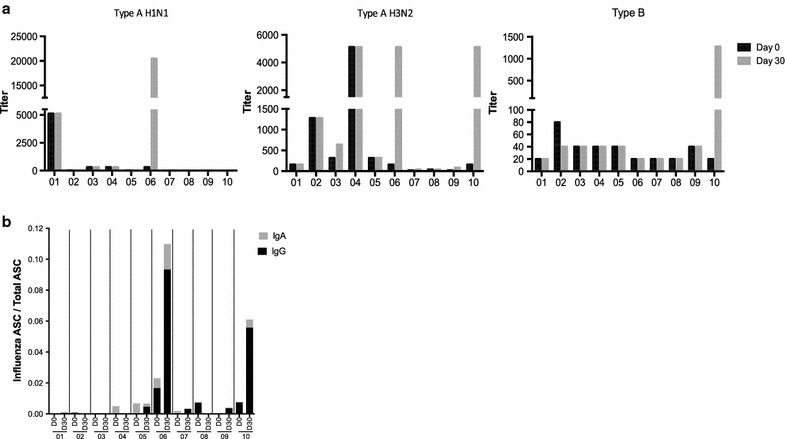
Impaired influenza-specific antibody production in AML patients who received influenza vaccination. **a** Viral-neutralizing antibody production was assessed through microneutralization assay. Day 0 titers indicated in* black*; day 30 titers indicated in *gray*. **b** Enumeration of antibody-secreting cells (ASC) through B-cell ELISPOT. IgA indicated in *gray*; IgG indicated in *black*

Only 2 of 10 of AML patients seroconverted (fourfold or higher antibody titer at day 30 compared to baseline) after vaccination to one or more of the influenza strains (AML responders, or AML-R) as assessed by microneutralization assay (Fig. 1a). One responder (AML 06) was 148 weeks post-chemotherapy, and the other (AML 10) had acute promyelocytic leukemia (APL). Some non-responders (AML-NR) had pre-existing titers but demonstrated no rise in neutralizing antibody titer after vaccination. These results were further confirmed using B-cell ELISPOT with the influenza vaccine formulation for 2012–2013. Patients 06 and 10 were the only two patients with influenza-specific IgA and IgG antibody secreting cells (ASCs) after influenza vaccination (Fig. 1b), and neither showed high levels of non-specific ASCs (Additional file 3: Figure S1).

### Global immunome assessment reveals differences between healthy subjects and AML patients after chemotherapy

**Fig. 2:**
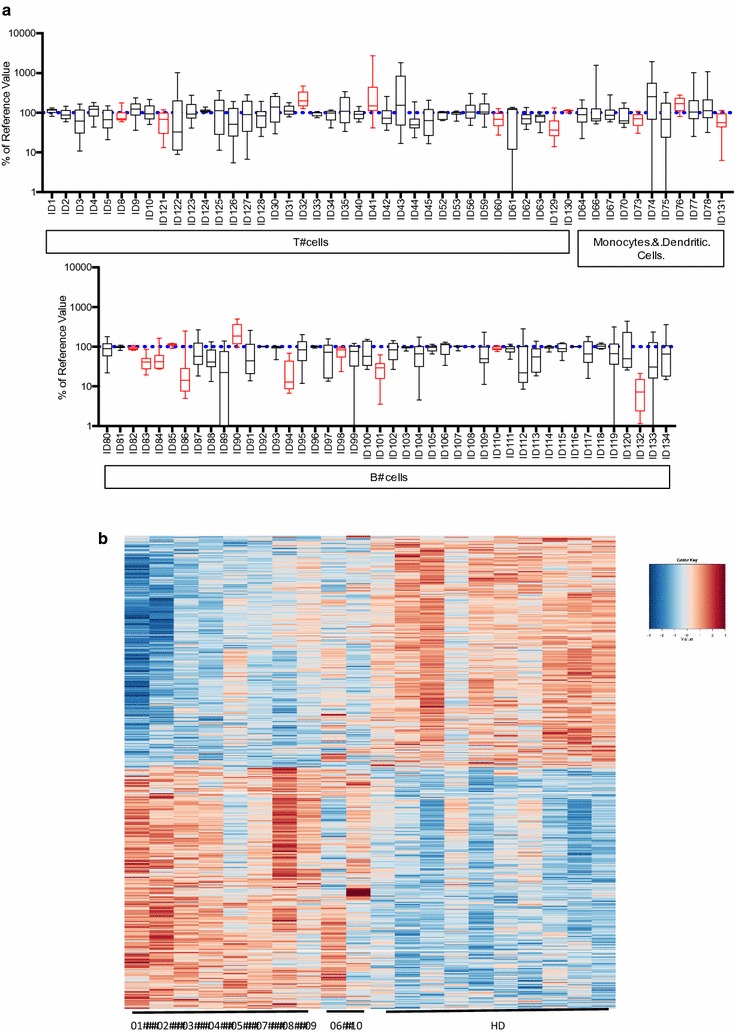
Global immunome analysis reveals differences between AML patients after chemotherapy and age/sex matched healthy donors. **a** *Box* and *whisker plots* of multi-parameter flow cytometry data. Frequencies of subpopulations T-cells, B-cells, dendritic cells, and monocytes were tabulated as a percentage of the average frequency of each cell population in HD. *Blue dotted line* indicates the normalized average in HD. *Red plots* mark populations where mean cell frequencies significantly (p < 0.05 with multiple testing correction) differed between AML (n = 10) and HD (n = 10). **b** Heat map generated from a supervised clustering of gene expression data. *Each column* represents an individual subject; *each row* represents a gene. First 8* columns* are AML-NR, next 2* columns* are AML-R, and last 10* columns* are HD. All data represents baseline gene expression. The genes were filtered using criteria of absolute value of log-fold-change higher than 0.2 and FDR-adjusted p value less than 0.05. Up- and down-regulated genes are noted by *colors* indicated in *color key*

To look for global differences between AML patients after chemotherapy (before influenza vaccination) and age- and gender-matched HDs, deep immunophenotyping of immune cell populations was conducted. In total, 93 cell populations were analyzed, listed in Additional file 2: Table S3. To better visualize global differences in cell population frequencies, we plotted the frequencies of the cell populations from the AML patients normalized to the average frequency seen in HD, which illustrates relatively over-expressed and under-expressed immune subsets (Fig. 2a). We identified 7/38 (18.4%) T-cell populations, 3/11 (27.3%) monocyte and dendritic cell populations, and 11/44 (25.0%) B-cell populations where the mean frequencies in AML (n = 10) and HD (n = 10) significantly differed from one another, highlighted in Fig. 2a. We also specifically focused on eight AML patients who did not respond to influenza vaccination (AML-NR) in comparison to HD and found 4/38 (10.5%) T-cell populations where the mean frequencies significantly differed, 3 of which overlapped with T-cell populations identified when considering all 10 AML patients. Two of 3 monocyte and dendritic cell populations also had significantly different mean frequencies in AML-NR vs HD. By these same metrics, we identified 10/44 (22.7%) B-cell populations where mean frequencies significantly differed between AML-NR and HD, 9 of which overlapped with B-cell populations identified from looking at all 10 AML patients. We also found 4/11 (10.5%), 4/11 (36.4%), and 7/44 (15.9%) T-cell, monocyte and dendritic cell, and B-cell populations, respectively, were the variances in cell frequencies between AML and HD significantly differed from one another. All data are summarized in Additional file 2: Table S4. Together, these data highlight heterogeneity across our AML cohort, a greater perturbation in the B-cell compartment in AML patients after chemotherapy, and cell populations that may be especially affected in AML patients who do not respond to influenza vaccination.

We next considered differences in global gene expression profiles to investigate any underlying genetic pathways potentially driving the responses we observed by analyzing microarray data derived from PBMCs of AML patients and HD. Supervised clustering between the eight AML-NR at baseline versus HD identified 1871 genes that were significantly differentially expressed, with 846 genes up-regulated and 1025 genes down-regulated in AML-NR (Fig. 2b). Gene set enrichment analysis (GSEA) of positively differentially expressed genes (DEG) in AML-NR revealed 111 gene sets with significant p-values (adjusted for multiple testing), including many immune-related, signaling, and cell death pathways (Additional file 2: Table S5, Additional file 3: Figure S2). Specifically looking at leukocyte development and activation-related gene sets revealed several genes that drove their positive regulation, including LGALS9 (Additional file 2: Table S6). We noted that the two AML responders had markedly different gene expression profiles from one another and from HD. GSEA analysis of the 240 genes that were upregulated in AML 06 versus HD revealed an upregulation of many of the same gene sets up-regulated in the AML-NR (Additional file 2: Table S7). The upregulation of genes related to the immune response, immune development and activation, cell signaling, and cell death in AML-NR indicate that despite irregularities in cell frequencies, the immune system in these patients is highly active and likely undergoing immune reconstitution, even in the patient (AML 06) who was in remission for 3 years at the time of enrollment to this study.

### Increased transitional B-cell and lower memory B-cell frequencies in AML patients after chemotherapy compared with healthy donors

**Fig. 3:**
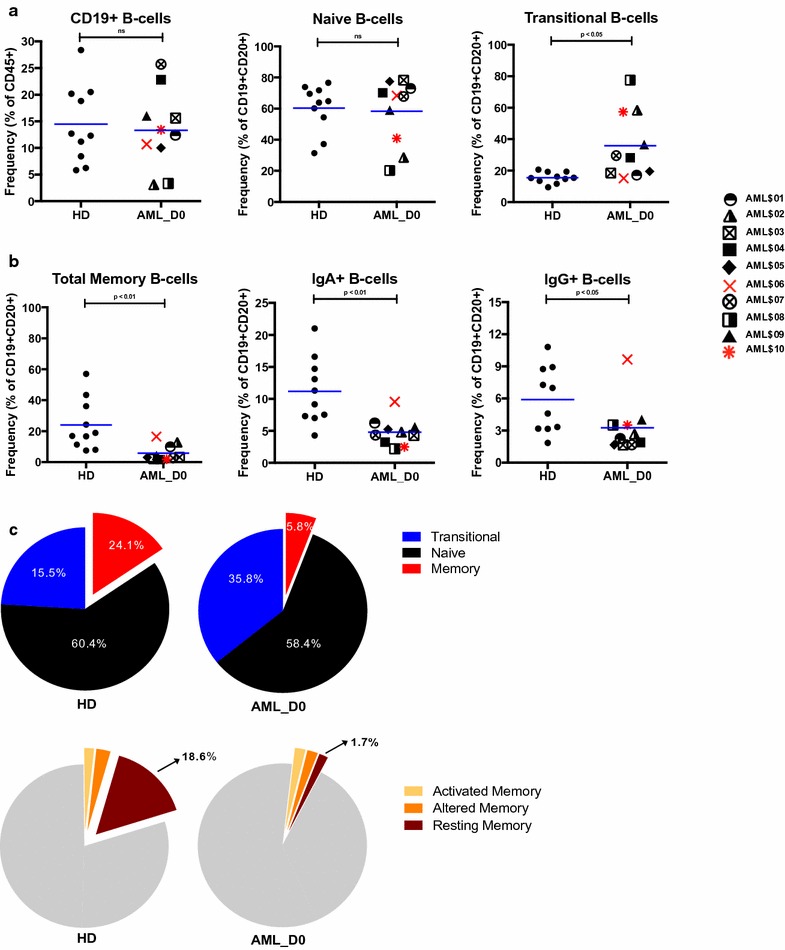
Greater frequencies of transitional B-cells and dramatically fewer memory B-cells in AML patients at baseline.** a** Frequencies of total CD19+ B-cells, naïve B-cells, and transitional B-cells in HD and AML at baseline and day 30.** b** Frequencies of total memory, IgA+, and IgG+ B-cells in HD and AML at baseline. AML patients indicated in *figure key*.** c** Proportions of transitional, naïve, and memory B-cells (*top pie charts*) and proportions of resting memory, altered memory, and activated memory B-cells (*bottom pie charts*) in HD and AML at baseline

We examined the frequencies of differing cell populations in greater detail, specifically considering both mean frequencies and variances in frequency distribution between AML and HD, to better characterize the data between groups. Frequencies of total CD19+ B-cells (ID80) were comparable between AML and HD (mean frequency ± SEM: 13.3% ± 7.3 vs. 14.5% ± 2.3, p = 0.8), and they ranged from 3.2 to 25.7% in AML and 5.9 to 28.4% in HD (Fig. 3a, left panel). Similarly, frequencies of naïve B-cells (ID106) in AML mirrored those seen in HD (58.4% ± 6.6 vs. 60.4% ± 4.8) with similar ranges (Fig. 3a, middle panel). However, there were significantly higher frequencies of transitional B-cells (ID90) in AML patients than in HD (35.5% ± 6.8 vs. 15.5% ± 6.7, p < 0.05) (Fig. 3a, right panel). The range of these cells was narrow in HD (9.0–20.7%) and strikingly wide in AML patients (15.2–77.6%), such that the variance significantly differed between HD and AML (p < 0.05). We saw a reduction of total memory B-cells (ID94) in AML at baseline compared to HD (5.8% ± 1.7 vs. 24.1% ± 5.2, p < 0.01) (Fig. 3b, left panel). This reduction was also mirrored in the mature B-cell IgA+ (ID83) (4.8% ± 0.7 vs. 11.2% ± 1.6, p < 0.01) and IgG+ (ID84) (3.2% ± 0.8 vs. 5.9% ± 1.0, p < 0.05) compartments (Fig. 3b, middle and right panels).

Figure 3c shows relative proportions of immature/transitional, naïve, and memory B-cells in all AML compared to HD, illustrating the abnormally high frequencies of immature/transitional and markedly low frequencies of memory B-cells. Consideration of some constituent subpopulations of the memory B-cell compartment (resting memory, altered or tissue-like memory, and activated memory) reveals that resting memory B-cell (ID132) frequencies in AML are significantly reduced, being less than 10% of frequency seen in HD (mean frequency ± SEM: 1.71% ± 0.45 vs. 18.5% ± 4.29) (Fig. 3c, bottom). Furthermore, the variance of frequencies of resting memory cells significantly differed between AML and HD (p < 0.05). We also noted reduced frequencies of CD86+ naïve (ID110) and memory B-cell populations (ID98) (Additional file 3: Figure S3) and several activated memory B-cell subsets with significantly different variances between AML and HD (summarized in Additional file 2: Table S4). We saw no significant differences between the means in any of these populations when comparing patients at baseline and 30 days post-vaccination (Additional file 3: Figure S4).

### Magnitude of B-cell subset abnormalities decline with increasing time elapsed since chemotherapy

**Fig. 4:**
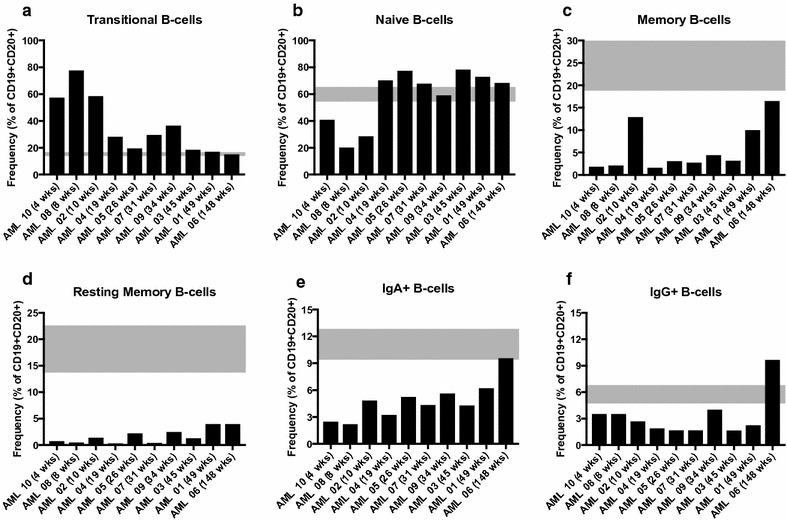
Recovery of B-cell populations in AML patients is correlated with increasing time since chemotherapy. AML patients at baseline are ranked by time since end of chemotherapy and B-cell frequencies plotted. **a** Transitional B-cells. **b** Naïve B-cells. **c** Total memory B-cells. **d** Resting memory B-cells. **e** IgA+ B-cells. **f** IgG+ B-cells. *Gray boxes* highlight mean values ± SEM of the HDs

As the time since the cessation of chemotherapy (or, the time since start of complete remission) varied across the 10 AML patients, we ranked these patients based on time elapsed since treatment and examined general trends of the B-cell populations over time, keeping in mind the heterogeneity of our cohort. Frequencies of transitional B-cells were lower in those patients sampled further from completion of chemotherapy (Fig. 4a). The three patients least removed from chemotherapy also had the lowest frequencies of naïve B-cells, suggesting rapid B-cell development in the 6 months after the cessation of treatment. Naïve B-cell frequencies were unchanged in patients 15 weeks or more post chemotherapy (Fig. 4b), while memory B-cells increased with time (Fig. 4c). Interestingly, frequencies of resting memory B-cells were well below the average ± SEM in HD for all AML patients (Fig. 4d). While mature IgA+ B-cell frequencies also increased with time elapsed since chemotherapy (Fig. 4e), mature IgG+ B-cell frequency remained consistent across all patients in the first year after chemotherapy, and these patients had fewer frequencies of IgG+ B-cells than the average ± SEM in HD (Fig. 4f). These trends indicate a return to healthy frequencies of transitional and naïve B-cells roughly 6 months after the end of chemotherapy but a more uneven recovery of memory and effector B-cell subsets over time.

### B-cell repertoire is diverse, but antigen-inexperienced, in AML patients after chemotherapy

To determine whether the B-cells from AML patients had molecular evidence of selection and mutation, we sequenced the B-cell receptor (BCR) complementarity-determining region 3 (CDR3) region of the immunoglobulin heavy (IGH) chain. There were no differences in the ratios of productive to non-productive rearrangements (86%:14% vs. 84%:16%) or in overall clonality (0.029 vs. 0.030) in AML compared to HD (Additional file 3: Figure S5). We next looked at IGH CDR3 length, as this characteristic is important in determining BCR diversity. CDR3 length is approximately normally distributed in HD and in AML, with a few exceptions. Patients sampled at early time points post-chemotherapy have greater variability in CDR3 length and exhibited a shorter CDR3 loop distribution. The remaining patients who were 19 weeks or greater after completion of chemotherapy have CDR3 length distribution that closely aligns with that seen in HD (Additional file 3: Figure S6).

**Fig. 5:**
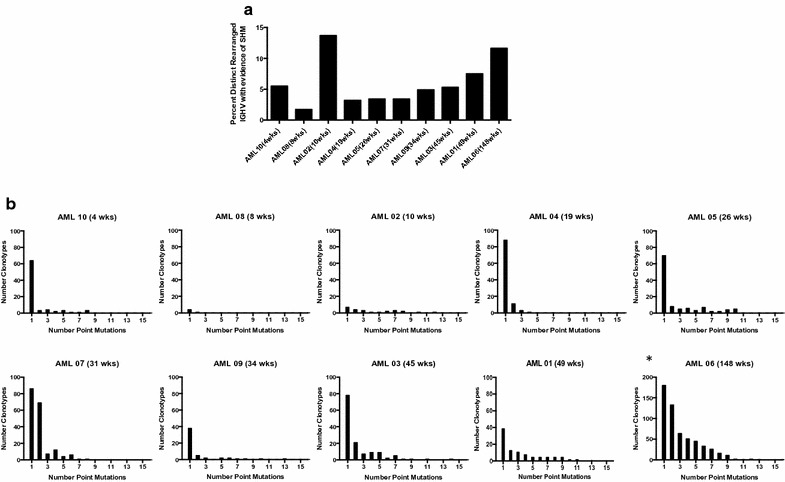
IGH CDR3 sequencing of AML patients at baseline suggest that B-cells are developing and antigen-inexperienced. **a** Frequencies of clonotypes with point mutations in their IGHV CDR3 regions as a result of SHM in AML patients ranked by time since end of chemotherapy. **b** The number of clonotypes with different numbers of point mutations in their CDR3 region were calculated for each patient. The *asterisk* indicates a different y-axis scale for AML patient 06

We considered the frequencies of distinct clonotypes with evidence of somatic hypermutation (SHM) in their IGH variable (IGHV) regions. We observed increased frequency of SHM in AML patients associated with time since last chemotherapy (Fig. 5a), and the percentage of distinct rearranged IGHV regions with evidence of SHM also roughly mirror the trends seen in the number of total memory B-cells (Fig. 4c). We delved deeper into the number of point mutations occurring in distinct clonotypes of B-cells of AML patients, and again noted that while AML patients least removed from chemotherapy had few to no CDR3 loops with more than one mutation, patients further into their remission demonstrated a greater number of point mutations in a greater number of clonotypes, indicative of somatic hypermutation (SHM) (Fig. 5b). Together, these data suggest that B-cell lymphopoiesis and affinity maturation begins after the cessation of chemotherapy, and the humoral immune system in these AML patients gradually increased the output of antigen-experienced memory B-cells in the first year of remission.

### AML patients after consolidation chemotherapy demonstrated generally recovered T-cell frequencies and function

**Fig. 6:**
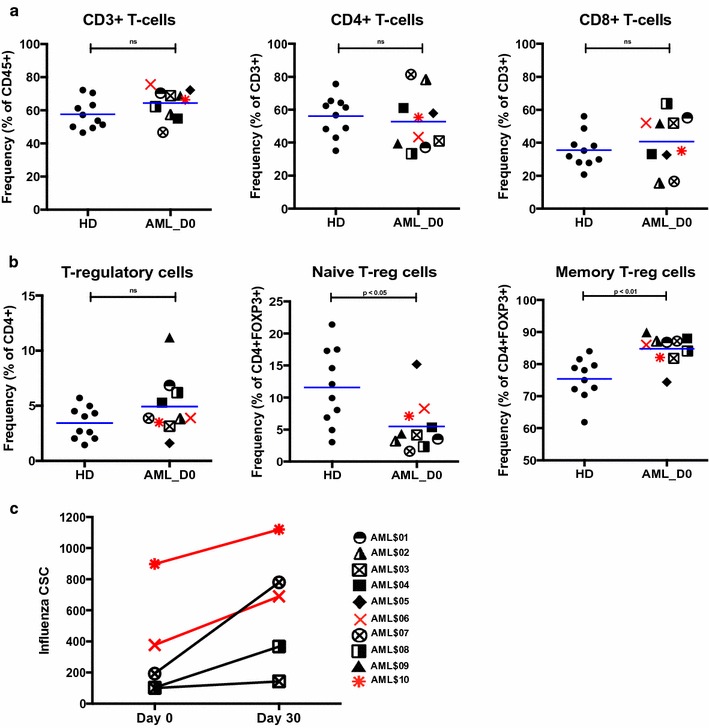
Recovered T-cell frequencies and function at baseline in AML patients. **a** Frequencies of total CD3+, CD4+ T-helper, and CD8+ T-cytotoxic cells in HD and AML baseline. **b** Frequencies of total CD4+ FOXP3+ T-regs, naïve T-regs and of memory T-regs in HD and AML baseline. Mean frequencies indicated by *blue lines*. **c** Influenza-specific cytokine secretion on day 30 versus baseline in 5 evaluable AML patients, including two responders (*red*) and three non-responders (*black*). AML patients indicated in *figure key*

AML patients in this study demonstrated no significant differences when compared to HD in frequencies (mean frequency ± SEM) of total CD3+ (ID1) (64.4% ± 2.8 vs. 57.5% ± 2.8), CD4+ (ID2) (52.8% ± 5.4 vs. 56.1% ± 3.8), or CD8+ (ID4) (40.7% ± 5.3 vs. 35.5% ± 3.3) T-cell populations (Fig. 6a). In addition, we observed no discernable changes or trends in any of these T-cell populations in AML patients between baseline and post-vaccination time points, nor when the patients were ranked by time elapsed since end of consolidation chemotherapy (Additional file 3: Figures S7, S8). The mean ratio of CD4+ to CD8+ T-cells for all 10 AML patients was 1.9–1, similar to the ratio seen in HD (1.8–1). Discrimination of CD4+ T-helper, and CD8+ T-cytotoxic into their naïve and memory subpopulations revealed no significant differences between the average frequencies seen in AML and HD, although the AML patients did demonstrate greater heterogeneity (Additional file 3: Figures S9, S10). Of note, we observed highly varied distributions of several cytotoxic CD8+ T-cell population frequencies in AML compared to HD (summarized in Additional file 2: Table S4).

We observed differences in many cell populations between AML and HD that include FOXP3+ T-regulatory cells (T-regs) (summarized in Additional file 2: Table S4). Frequencies of total T-regs (ID59) were similar between AML and HD (mean frequency ± SEM: 4.9% ± 0.8 vs. 3.4% ± 0.5) (Fig. 6b, left panel). However, AML patients did have significantly reduced frequencies of naïve T-regs (ID129) (5.5% ± 1.3 vs. 11.6% ± 1.9, p < 0.05) and higher frequencies of memory T-regs (ID130) (84.8% ± 1.4 vs. 75.4% ± 2.0, p < 0.01) (Fig. 6b, middle and right panels) compared to HD.

We saw much variation but no discernable pattern in the over- or under-expressed frequencies of monocyte and dendritic cell populations between AML and HD (Fig. 2a; Additional file 2: Table S4). However, we did observe that the frequencies of conventional or myeloid dendritic cells (mDCs) (ID73) were lower in AML than in HD, while the frequencies of plasmacytoid dendritic cells (pDCs) (ID76) were higher (Additional file 3: Figure S11).

In addition to deep phenotypic assessment, we also directly assessed the functional capacity of T-cells in AML using IFNγ T-cell ELISPOT assays in 5 patients who had available baseline and post-vaccination samples (including the 2 AML-R and 3 AML-NR). Surprisingly, all 5 patients saw increases in influenza-specific cytokine secretion after influenza vaccination (increases of 1.24- to 4.0-fold on day 30 over day 0) (Fig. 6c). Together, our data suggests that despite deficiencies in humoral immunity and apparent reduction of mDC frequencies, the T-cell compartment in AML patients has recovered as soon as 1 month post-consolidation and can generate a functional cellular immune response.

### Cell surface immune checkpoint expression is similar between AML patients in remission after chemotherapy and healthy donors

**Fig. 7:**
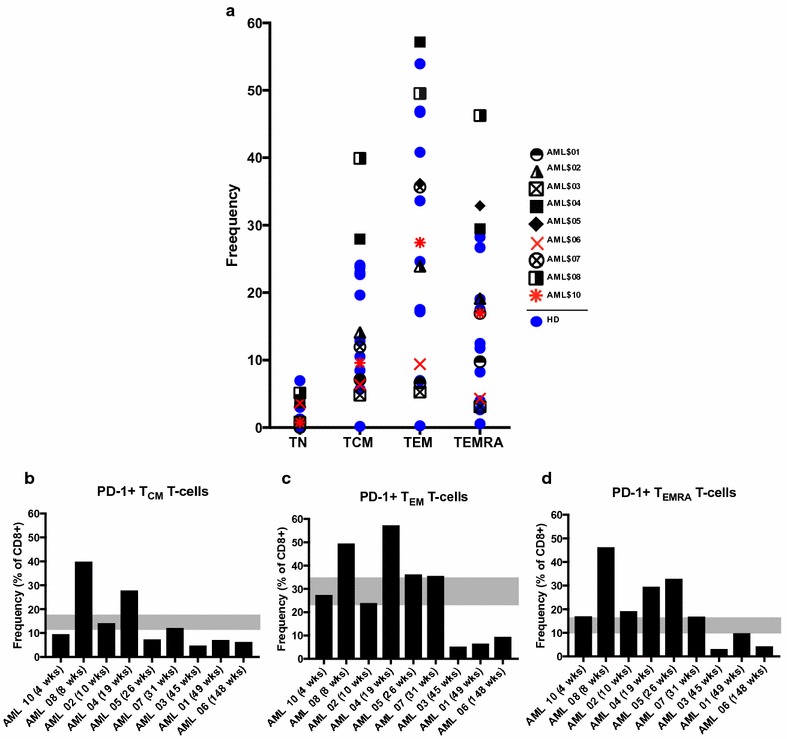
PD-1 expression on CD8+ T-cell subsets is similar between HD and AML at baseline. **a** Frequencies of PD-1+ total CD8+, naïve (TN), central memory (TCM), effector memory (TEM), and terminal effector (T_EMRA_) CD8+ T-cell populations. AML patients and HD indicated in *figure key*. **b**–**d** AML patients at baseline are ranked by time since end of chemotherapy and frequencies of indicated PD-1+ T-cell populations plotted. *Gray boxes* highlight mean values ± SEM of the HDs

Finally, as our protocol was designed to study a cohort of AML patients who may be considered as candidates for maintenance immunotherapies to prevent relapse, we sought to assess expression of cell surface immune checkpoint markers, namely, PD-1, TIM3, CTLA4, and 4-1BB in AML at baseline and in healthy donors on various CD8+ T-cell populations. We found no differences in overall immune checkpoint marker expression, including double positive expression of these markers, between AML at baseline and HD on any CD8+ T-cell population. Of particular interest is PD-1 expression, and in AML and HD, respectively, we saw similarly varied frequencies (mean frequency ± SEM) of PD-1+ total CD8+ T-cells (16.6% ± 4.6 vs. 13. 0% ± 2.7), CD8+ central memory (14.3% 4.0 vs. 15.1% vs. 2.7), CD8+ effector memory (27.9% ± 6.2 vs. 28.9% ± 5.8), and CD8+ terminal effector (19.9% ± 4.7 vs. 13.1% ± 3.1) (p > 0.05 for all) (Fig. 7a). Considering the AML patients based on time elapsed since treatment revealed that PD-1+ T-cells appeared to decrease in frequency with increasing time since chemotherapy (Fig. 7b–d).

## Discussion

While immune system reconstitution after chemotherapy is important from an infectious disease standpoint, understanding the kinetics and biology behind this phenomenon is also important in the context of anti-cancer immunotherapy to prevent relapse and maintain remission in patients. Our goal with this study was to perform a deep assessment of the state of the adaptive immune system in AML patients in a first clinical remission after consolidation chemotherapy, not only to further the body of evidence for the timing of vaccinations against infectious disease in these patients, but also to characterize patients who may be considered candidates for post-chemotherapy immunotherapy. To do this, we sought to explore patterns of immune reconstitution following consolidation chemotherapy in AML patients in remission and to look for indications of a functional response, or lack thereof, to vaccination. Our approach, when compared to previous studies, was unique in that we established a comprehensive picture of the state of the adaptive “immunome” by simultaneously examining the genetic, phenotypic, and functional consequences of chemotherapy in AML patients. Importantly, we show a dramatic impact in the B-cell compartment, which appears slower to recover than the T-cell compartment after AML chemotherapy. We further show that the inability of AML patients to produce protective antibody titers in response to influenza vaccination is likely due to multiple B-cell abnormalities in this cohort of patients, including increased frequencies of transitional B-cells, a lack of affinity-matured, class-switched B-cells, and an antigen-inexperienced B-cell repertoire. Of particular interest are the generally recovered T-cell frequencies and the cytokine-secretion functionality of CD8+ T-cells in response to influenza vaccination, suggesting that the two arms of the adaptive immune system are not equal in how they are affected by, or recover from, AML chemotherapy.

A recent study of post-vaccination responses to seasonal and pandemic influenza vaccination identified several immune subsets that were predictive of a robust and specific antibody response [17]. Among these predictive subsets, several CD38+ B-cell populations (ID91, ID96, ID103, ID108) were very highly correlated with a strong antibody response to influenza vaccination. However, in our data set, we saw no differences in the frequencies of these CD38+ populations in AML patients compared to HD, nor did we see any difference in their frequencies between the AML-NR and AML-R. The lack of a relationship between these populations and response to vaccination in our AML cohort and HD indicate that applying a signature to predict post-vaccination responses is difficult in patients who receive chemotherapy, likely due to fluctuating immune parameters as immune reconstitution occurs. T-cell responses may be better surrogates of response to vaccination, especially in elderly individuals, but again, it has not been determined if this holds true in AML patients after they have received chemotherapy [26].

Previous studies in solid tumors and in lymphoid hematological malignancies have reported a delayed B-cell recovery and function during and in the year following allo-HSCT, cytotoxic chemotherapy, or B-cell depleting agents [27–29]. In a small cohort of pediatric AML patients either still receiving or having just completed treatment, it was shown that chemotherapy appeared especially deleterious for naïve and memory B-cells [28]. However, it remained unknown whether these effects would be the same in adult AML patients specifically in a complete remission post-consolidation. Furthermore, while both cellular and humoral immune reconstitution has been characterized in the allo-HSCT setting [29–35], it remained unclear what immune deficiencies could be ascribed to the consolidation chemotherapy patients received to achieve a CR before they receive conditioning therapy before transplant.

In our study, the total frequencies of CD19+ B-cells were similar between AML patients before vaccination and HD. The increased frequency of circulating immature, transitional B-cells suggest higher output by the bone marrow of these cells to repopulate the humoral immune niche [29], and the near absence of memory B-cell subsets and class-switched B-cells suggests either an inherently increased susceptibility of both memory and effector B-cells to chemotherapy, an insufficient amount of time to fully recover these cell populations, or both. CD27+ memory B-cells, and especially resting memory B-cells, are responsible for generating antibody responses, and circulating influenza-specific memory B-cells can last for decades [36–39]. The consistent finding of a reduction of memory B-cells in all the AML patients implicates this loss in the poor responses observed to influenza vaccination and suggests that humoral immunity reconstitution is a very long process.

The large number of differentially up-regulated genes in the 8 AML patients who did not respond to influenza vaccination compared to HD points towards a very active cell machinery across many cell pathways in various immune cell subsets. Furthermore, gene set enrichment analyses with very stringent criteria produced a very large number of up-regulated gene sets, underscoring the magnitude of perturbation in the intracellular state of PBMCs from the AML patients. The over-expression of genes related to cell death, metabolism, and genes more generally related to the immune response are strongly suggestive that the immune system is subject to lymphopoiesis and reconstitution in AML patients. Of particular interest is the upregulation of galectin-9 (LGALS9), which is the ligand for TIM-3 [40]. LGALS9 can exert pleiotropic effects through TIM-3, and it has been shown to promote leukemic stem cell renewal, suggesting that this gene that appears to be up-regulated by the process of immune reconstitution after chemotherapy may also have a role in AML relapse [41, 42].

Since the loss of memory B-cells is a central finding of our work, we sought to further interrogate the status of these cells through B-cell receptor IGH chain repertoire sequencing. The sequencing data shows that AML patients after chemotherapy have the same success rate at forming productive sequences as HD and can eventually form BCRs against specific antigen. Interestingly, the percentage of rearranged IGHV regions with point mutations indicative of somatic hypermutation in each patient tracked with the total frequency of memory B-cells, which were highest in frequency in patients furthest from last chemotherapy. Although we did not specifically look at B-cells involved in germinal center reactions as we did not sample peripheral lymphoid tissue in this study [43], our findings show that the humoral immune system in AML patients after chemotherapy is still capable of generating B-cell memory.

Frequencies of various T-cell subpopulations were grossly normal in our cohort of AML patients. The relatively recovered and intact T-cell immune system in these patients and the ability of CD8+ T-cells to secrete IFNγ in an antigen-specific manner suggest that T-cell mediated immunotherapies are perhaps better suited at preventing and treating relapsed and refractory AML. We noted increased PD-1 expression on effector and memory T-cells shortly after the completion of chemotherapy in some of our AML patients. We believe there may be a window for immunotherapeutic intervention using immune checkpoint blockade to target residual disease in patients in remission. We do not fully address if the seemingly intact T-cell compartment in the blood of our patients were thymically derived or if surviving cells underwent peripheral expansion after chemotherapy. Recovery of CD4+ T-cells is thought to be generally thymus-independent, and one piece of corroborating evidence in our cohort lies in the higher frequency of memory T-regs and the reduced frequency of naïve T-regs in the AML patients, suggesting a proliferation of memory T-regs in the periphery as has been reported in the month immediately following chemotherapy [15, 44]. However, the inverse has been reported in other hematological malignancies [45, 46], which has been ascribed to a reduced susceptibility of naïve T-regs to oxidative stress [47]. While T-regs are important in the maintenance of B-cell homeostasis and tolerance [48] in mice models receiving influenza vaccination, CD4+ FOXP3+ T-regs had no effect on B-cell responses [49]. However, this same study reported increased frequencies of T-regs in response to influenza vaccination, but we did not observe any appreciable increase in the frequencies of naïve or memory T-regs in our AML cohort after vaccination [49]. Thus, our observations on the relative frequencies of T-reg subsets in AML patients after chemotherapy differ somewhat with what has been reported in the literature, and more work into the underlying mechanisms of T-reg reconstitution and responses in the period immediately following chemotherapy and its effects on the success of non-transplant immunotherapeutic strategies is warranted.

A limitation of our work is the exclusion of CD161 and ICOS in our T-cell phenotyping panels, as recent reports identified populations of CD4+ CD161+ T-cells and ICOS+ CD38+ T-follicular helper cells that clonally expanded and persisted years after influenza vaccination in AML patients receiving chemotherapy and in healthy individuals receiving successive influenza vaccinations, respectively [50, 51]. Additionally, the flow cytometric panels used in this study did not include natural killer (NK) cells. As a previous report identified recovered NK cell frequencies in an AML patient in remission [52], future planned studies will examine the frequencies of NK cells in AML patients in remission, as this cell population is an active area of exploration as an anti-leukemic immunotherapy [53]. Additionally, we will compare T-cells in the marrow compartment with those sampled from the blood to examine whether there are phenotypic and functional differences between cells from these two related yet distinct sites of disease. Furthermore, one consideration we have not addressed is the possibility of AML minimal residual disease (MRD) under the threshold of detection by morphologic examination contributing to local immunosuppression in the tumor microenvironment and its effect on lymphocyte recovery. It has been shown that AML blasts can induce IL-10 and IL-17 production by Th17 cells [54], and IL-10 can have differential effects on B-cells depending on their differentiation and activation status [55]. The effect of AML MRD on B-cell recovery in particular remains to be determined, and studying Th17 cells as well in the context of AML remission is also warranted.

While our study was small and our cohort of AML patients was diverse in terms of disease etiology, clinical outcomes, and duration into their first remission, we strikingly found similar patterns of immune dysfunction across all the patients. Notably, when we ranked the patients based on time elapsed since chemotherapy and used cell frequency trends as surrogates for immune reconstitution over time, the degree of dysfunction was less in those patients that were further away from chemotherapy. Our better understanding of the changes in adaptive immune cell subsets after chemotherapy in AML patients will be useful in designing immunotherapies that can work with existing immune capacity to eliminate residual disease and maintain a remission in patients with AML.

## Conclusions

In conclusion, we have shown that impaired responses to influenza vaccination in our AML patients is associated with altered B-cell composition and impaired B-cell immunity. Successful responses to vaccinations require both functional cellular and humoral adaptive immunity, and the uncoupled B-cell and T-cell responses we have described have implications for the success of certain immunotherapies. These findings may have implications for the use of immunotherapy in AML.

## Additional files

**Additional file 1.** Supplemental methods.

**Additional file 2.** Supplemental tables.

**Additional file 3.** Supplemental figures.

## Abbreviations

AML: acute myeloid leukemia
CR: complete remission
CR1: first complete remission
Allo-HSCT: allogeneic hematopoietic stem cell transplantation
HD: healthy donor
PBMCs: peripheral blood mononuclear cells
CSCs: cytokine-secreting T-cells
ASCs: antibody-secreting B-cells
APL: acute promyelocytic leukemia
DEG: differentially expressed gene
SEM: standard error of the mean
BCR: B-cell receptor
IGH: immunoglobulin heavy
IGHV: immunoglobulin heavy chain variable
CDR3: complementarity-determining region 3
SHM: somatic hypermutation
T-regs: T-regulatory cells
