# Supplementary material for: Impaired B cell immunity in acute myeloid leukemia patients after chemotherapy
Source: J Transl Med. 2017 Jul 10;15:155. doi: 10.1186/s12967-017-1252-2 (PMC5504716; doi:10.1186/s12967-017-1252-2)
Supplement: Supplementary file 1 — Additional file 1. Supplemental methods. [file 12967_2017_1252_MOESM1_ESM.pdf]

Additional file 1: Supplemental Methods

Nucleic acid extraction

Total RNA, including small RNA, was isolated from HD and AML PBMCs using the miRNeasy Mini Kit (Qiagen) according to manufacturer's protocol. RNA quantity and quality was measured using a Nanodrop 8000 Spectrophotometer (Thermo Scientific). Degradation of RNA (as measured by RNA integrity number, or RIN) was also assessed by running samples on an Agilent 2100 Bioanalyzer (Agilent Technologies). DNA was isolated from PAXgene blood RNA tubes using a modified protocol detailed in supplemental methods. DNA quantity and quality was measured using a Nanodrop 1000 Spectrophotometer.

T-cell Enzyme-linked immunosorbent spot (ELISPOT) assay

All T-cell ELISPOT assays were performed at the Laboratory of Cell-Mediated Immunity at Leidos Biomedical, Inc. (formerly SAIC-Frederick, Inc.) which is certified by Clinical Laboratory Improvements Amendments (CLIA). Two frozen normal donor controls with known response values were run with each assay to assure quality control of the assay results. For all assays, at least one of the two controls was within 2SD of the lab-generated means for CEF (23 peptides from MHC class I-restricted T-cell epitopes from human CMV, EBV and influenza virus, designed to stimulate T cells from donors with a variety of HLA types (Mabtech). All assays were performed on 300K or 100K PBMC in ELISPOT media containing RPMI (Gibco), 5% human AB serum (Gemini Bio-Products, West Sacramento, CA, USA), 1% Pen/Strep-L-glutamine (Gibco) and 2.5% HEPES (Gibco). Briefly, the day before assay setup, 96-well polyvinylidene fluoride (PVDF) membrane and HTS opaque plates (Millipore) were coated overnight with a 1:100 dilution of anti-human IFN-γ capture antibody (1mg/mL, Mabtech) in DPBS at room temperature. Antibody-coated plates were washed four times in DPBS the next day and blocked with 5% human AB ELISPOT medium at 37°C for approximately 2 hours. The PBMCs were stimulated under the following conditions: cells alone, 1ug/mL FluZone (Sanofi-Pasteur), 2ug/mL influenza MP1 peptide pool, 2ug/mL CEF peptide pool, or with

2.5ug/mL PHA. The cells were incubated for 18-20 hours at 37°C and 5% CO2. The next day, the plates were manually washed six times with 0.05% Tween 20 in DPBS, followed by a 2-hour incubation at room temperature with a 1:2000 dilution of the biotinylated secondary antibody, anti-human IFN-γ (1 mg/mL Mabtech) in DPBS/1% bovine serum albumin/0.05% Tween. After incubation and four washes in DPBS to remove excess antibody, a 1:3000 dilution of streptavidin alkaline phosphatase (Mabtech) in DPBS/1% bovine serum albumin, was added to each well for 1 hour at room temperature followed by 4 manual washes in DPBS. Finally, the BCIP/NPT substrate, 100 ul/well (KPL) was added for 7-10 minutes, resulting in the development of spots. The reaction was stopped by washing three times in distilled water. Plates were dried overnight and the spots were visualized and counted using the ImmunoSpot Imaging Analyzer system (Cellular Technology). ELISPOT results were expressed as the "number of spots per 10^6 responder cells" after subtracting background spots obtained in wells of PBMC alone. When available, PBMC from day 0 and day 30 were analyzed in the same assay to avoid inter-assay variability

B-cell Enzyme-linked immunosorbent spot (ELISPOT) assay

For B-cell ELISPOTs, mature CD10- B-cells were isolated from PBMCs through negative selection using a B-cell enrichment cocktail and magnetic bead-based robotic separation (StemCell Technologies). These B-cells were plated and incubated with influenza vaccine formulation for 2012-2013 (Sanofi-Pasteur), the polyclonal activators *Staphylococcus aureas* Cowan (SAC; EMD Biosciences) and CpG oligodeoxynucleotide (Operon), or with control keyhole limpet hemocyanin control antigen (EMD Calbiochem). After plates were washed and incubated with substrate, spots were visualized and counted with the ImmunoSpot Imaging Analyzer System. The number of ASCs was adjusted based on background in control wells and number of input cells.

DNA extraction from blood RNA PAXgene tubes

DNA was extracted from PAXgene tubes containing whole blood using a protocol adapted from manufacturer's instructions (Qiagen). PAXgene tubes were thawed at RT for 2 hours, and 4ml of the

suspension was transferred to a 15ml conical tube, which was centrifuged at 3000rpm for 10 minutes. The supernatant was removed and cell pellet resuspended in 5ml H_20, which was then centrifuged at 3000rpm for 10 minutes. The supernatant was removed and cell pellet resuspended in 200ul buffer BR1, and the entire solution was transferred to a 1.5ml Eppendorf tube containing 20ul kit-provided protease and 4ul RNaseA (at a concentration of 100mg/ml). Next, 200ul Buffer AL was added and the solution vortexed for 15 seconds. The tubes were incubated for 10 minutes at 56°C and then centrifuged for 3 minutes at 13,000rpm. Resulting supernatant was transferred to a new 1.5ml Eppendorf tube, and 300ul 100% ethanol was added. The solution was pulse vortexed. The solution was then transferred to a Qia mini blood column and centrifuged at 8,000rpm for 1 minute. The columns containing DNA were then washed with 500ul Buffer AW1 and then 500ul Buffer AW2 (with a new collection tube each time). The columns were dried by centrifuging at 13,000rpm for 1 minute. Finally, 140ul Buffer TE was added to the column membrane for 7 minutes, and then the column was centrifuged at 10,000rpm for 1 minute to elute DNA.

Deep sequencing of the B-cell receptor heavy chain – extended.

Specifically, the first PCR consists of forward and reverse amplification primers specific for every V and J gene segment and amplifies the hypervariable CDR3 of the immune receptor locus. The second PCR adds a proprietary barcode sequence and Illumina adapter sequences. CDR3 libraries were sequenced on the Illumina platform according to the manufacturer's instructions. Raw Illumina sequence reads were demultiplexed according to Adaptive Biotechnologies' proprietary barcode sequences. The resulting 130-base pair fragment is sufficient to assign the V(N)D(N)J genes spanning each unique CDR3 and translate to the amino acid sequence. V, D and J gene definitions were based on annotation in accordance with the IMGT database (www.imgt.org). The set of observed biological CDR3 sequences were subsequently normalized to correct for residual multiplex PCR amplification bias and quantified against a set of synthetic CDR3 sequence analogues[25]. The resulting data is filtered and clustered using both the relative frequency ratio between similar clones and a modified nearest-neighbor algorithm, to merge closely related sequences

and remove both PCR and sequencing errors, and any remaining contaminants. Data was analyzed using the ImmunoSEQ analyzer toolset.

Immune checkpoint flow cytometry

PD-1 staining was carefully optimized using a full-length PD-1 transfected CHO cell line as a positive control. Cell suspensions were incubated with with Human TruStain FcX block (Biolegend) for 10 minutes at room temperature, a 1:10,000 dilution of Zombie Green Viability Stain (Biolegend) reconstituted in DMSO and diluted in PBS for 10 minutes at room temperature, and finally the following antibody mix for 30 minutes in the dark at room temperature: BV421 CCR7 (G043H7), BV605 TIM-3 (clone F38-2E2), BV650 CD45RA (clone HI100), FITC CD14 (clone M5E2), FITC CD19 (clone HIB19), PE-Cy5 CD69 (clone FN50), PE-Cy7 4-1BB (clone 4B4-1), APC CTLA4 (clone L3D10), APC 750 Fire CD8 (clone SK1) (Biolegend), and PE PD-1 (clone MIH4) (eBioscience). Cells were fixed in a 1% paraformaldehyde solution for 20 minutes at room temperature, washed, stored overnight in the dark at 4 degrees, and acquired. Isotype controls for all immune checkpoint markers and a fluorescence-minus-one (FMO) control for PD-1 were included to best determine accurate gates around positive populations.

Acquisition was performed using a Becton Dickinson FACSAria Fusion (BD) equipped with FACSDIVA software (BD). Post-acquisition analysis was performed using FlowJo version 9.8.5 (Treestar, Inc.). Cell debris, doublets, CD14+, and CD19+ cells were excluded from analysis, and all subsequent T-cell populations were gated from live CD3+ events. Cell population frequencies were calculated as a percent of a parent population. Naïve (T_N) (CCR7+CD45RA+), central memory (T_{CM}) (CCR7+CD45RA-), effector memory (T_{EM}) (CCR7-CD45RA-), and terminal effector (T_{EMRA})(CCR7-CD45RA+) CD8+ T-cell populations were gated, and expression of PD-1, TIM-3, CTLA4, and 4-1BB was analyzed on these populations.
