# Supplementary material for: Impaired B cell immunity in acute myeloid leukemia patients after chemotherapy
Source: J Transl Med. 2017 Jul 10;15:155. doi: 10.1186/s12967-017-1252-2 (PMC5504716; doi:10.1186/s12967-017-1252-2)
Supplement: Supplementary file 3 — Additional file 3. Supplemental figures. [file 12967_2017_1252_MOESM3_ESM.pdf]

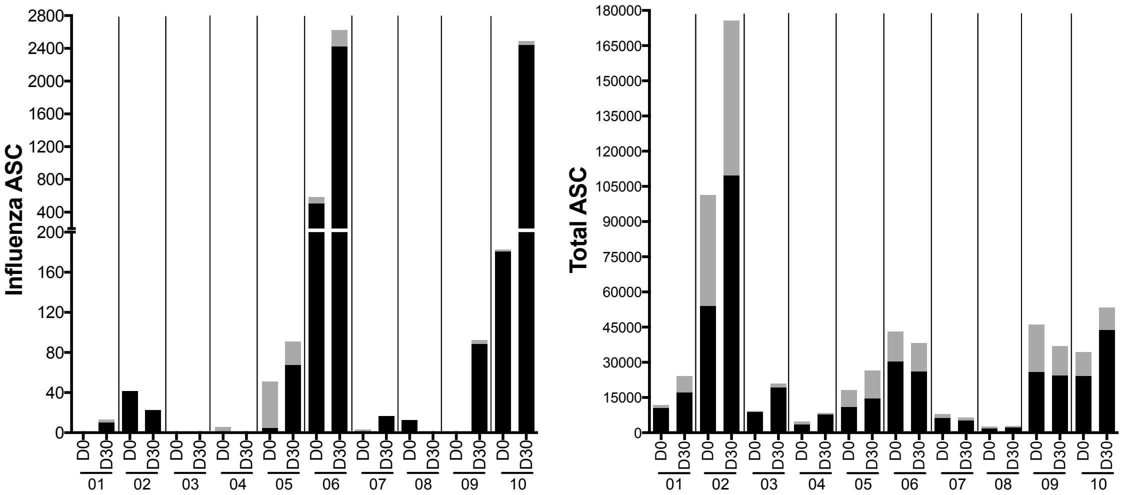

Fig. S1. Number of influenza-specific antibody-secreting cells (ASC) and total ASCs.

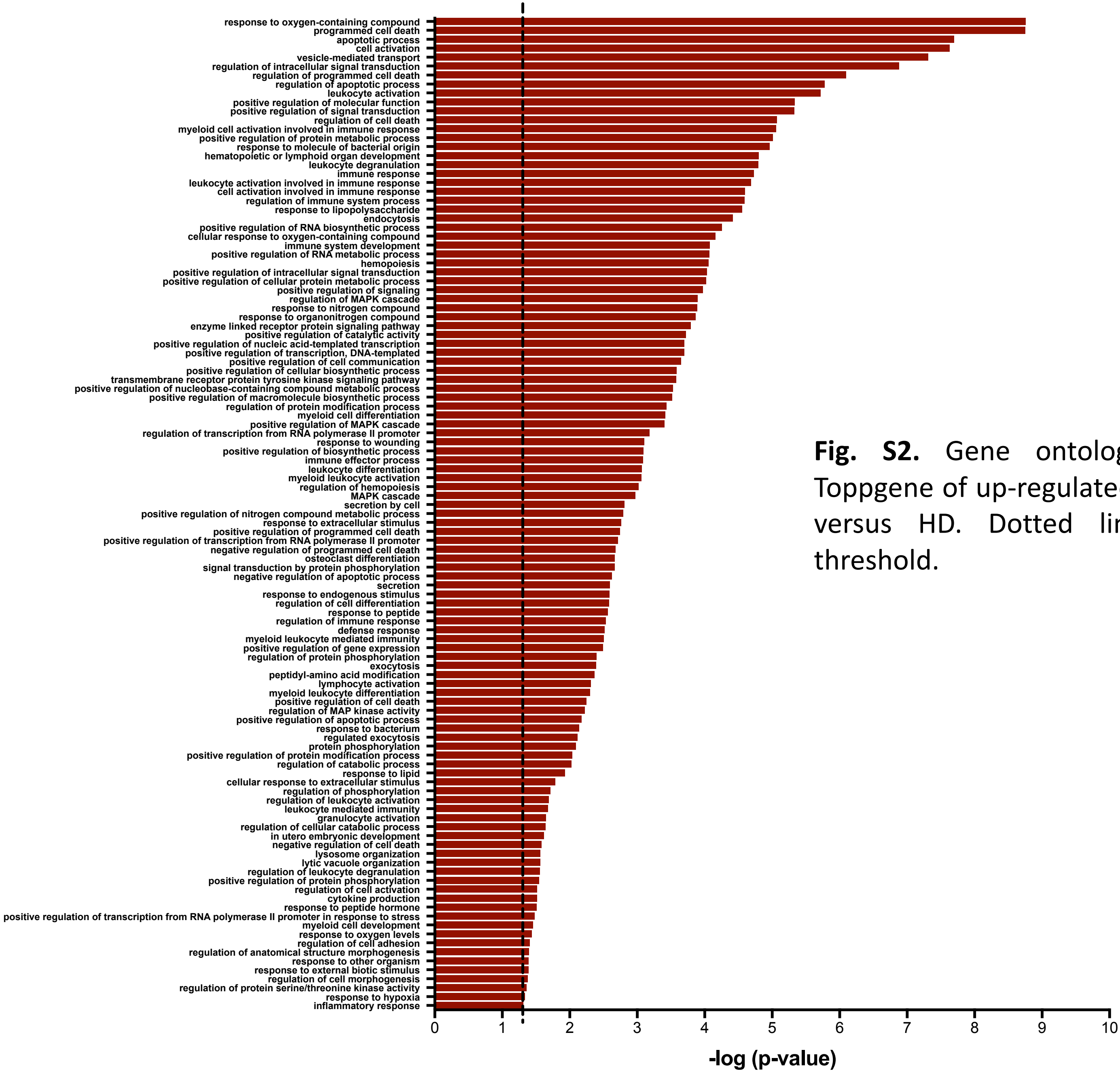

Fig. S2. Gene ontology analysis using Toppgene of up-regulated genes in AML-NR versus HD. Dotted line marks p=0.05 threshold.

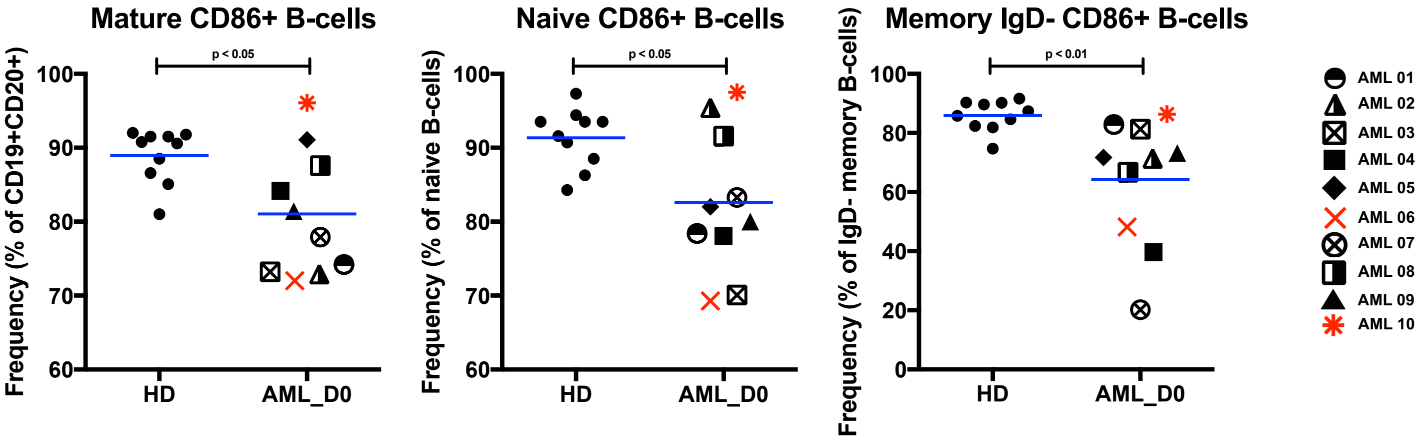

Fig. S3 Frequencies of CD86+ B-cell populations in HD and AML at baseline. AML patients are indicated in the figure key. Blue lines indicate average frequencies for each indicated group.

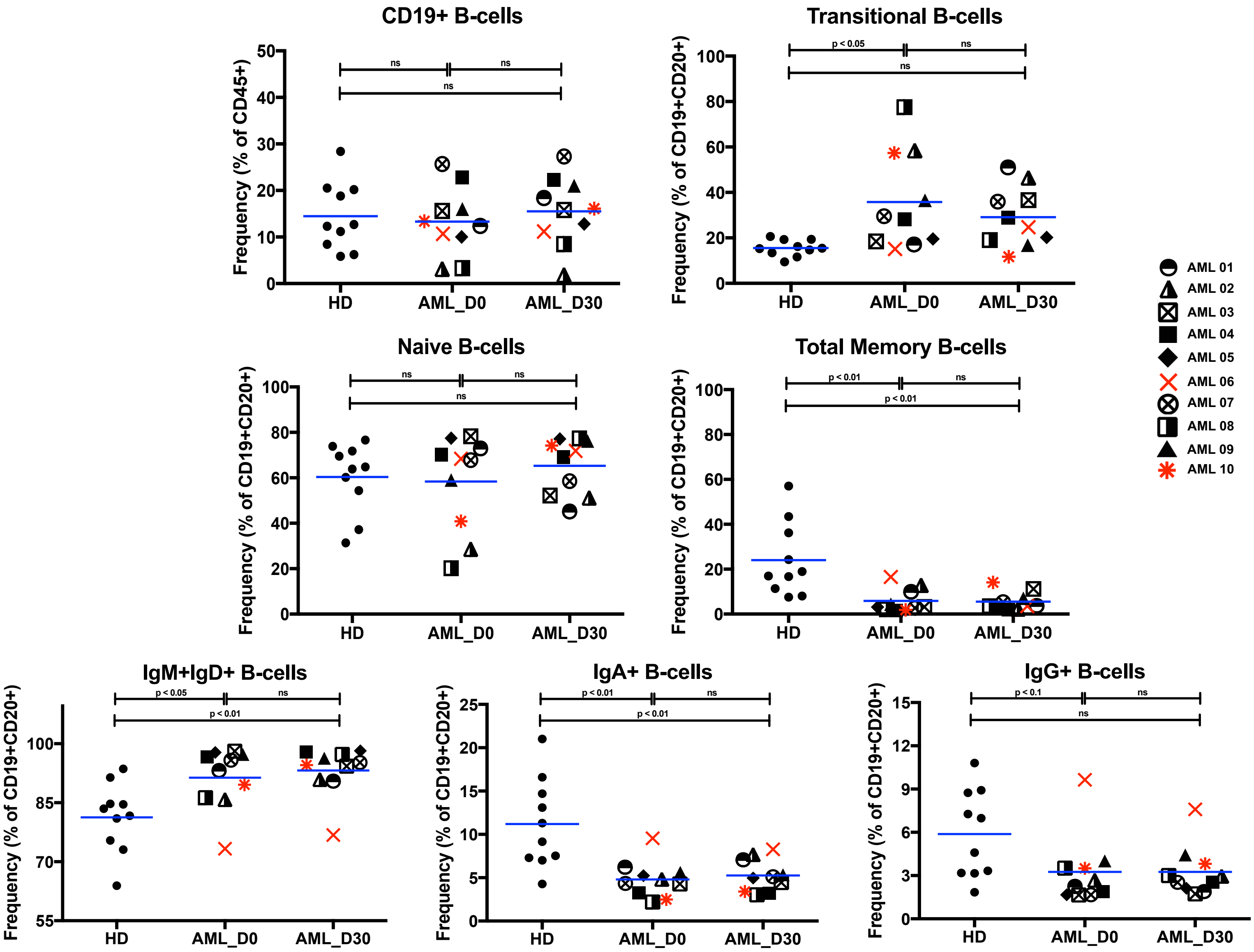

Fig. S4. Frequencies of B-cell populations in HD and AML at baseline and day 30. AML patients are indicated in the figure key. Blue lines indicate average frequencies for each group; “ns” indicates a non-significant difference.

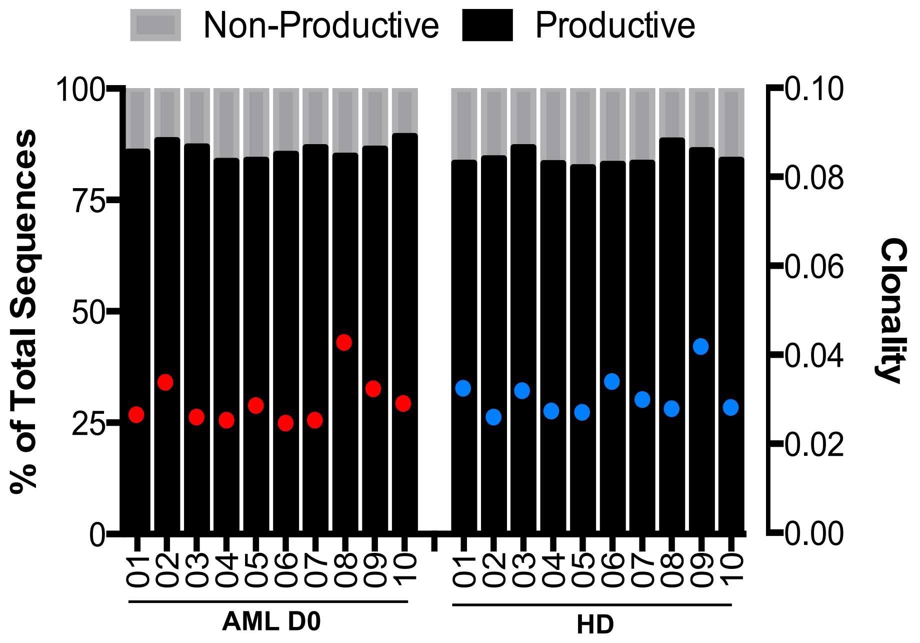

Fig. S5. Percentages of productive (black) and non-productive (gray) rearrangements in AML at baseline and 10 healthy individuals (different than matched HD used previously). Clonality in AML patients is indicated by red dots; clonality in HD indicated by blue dots.

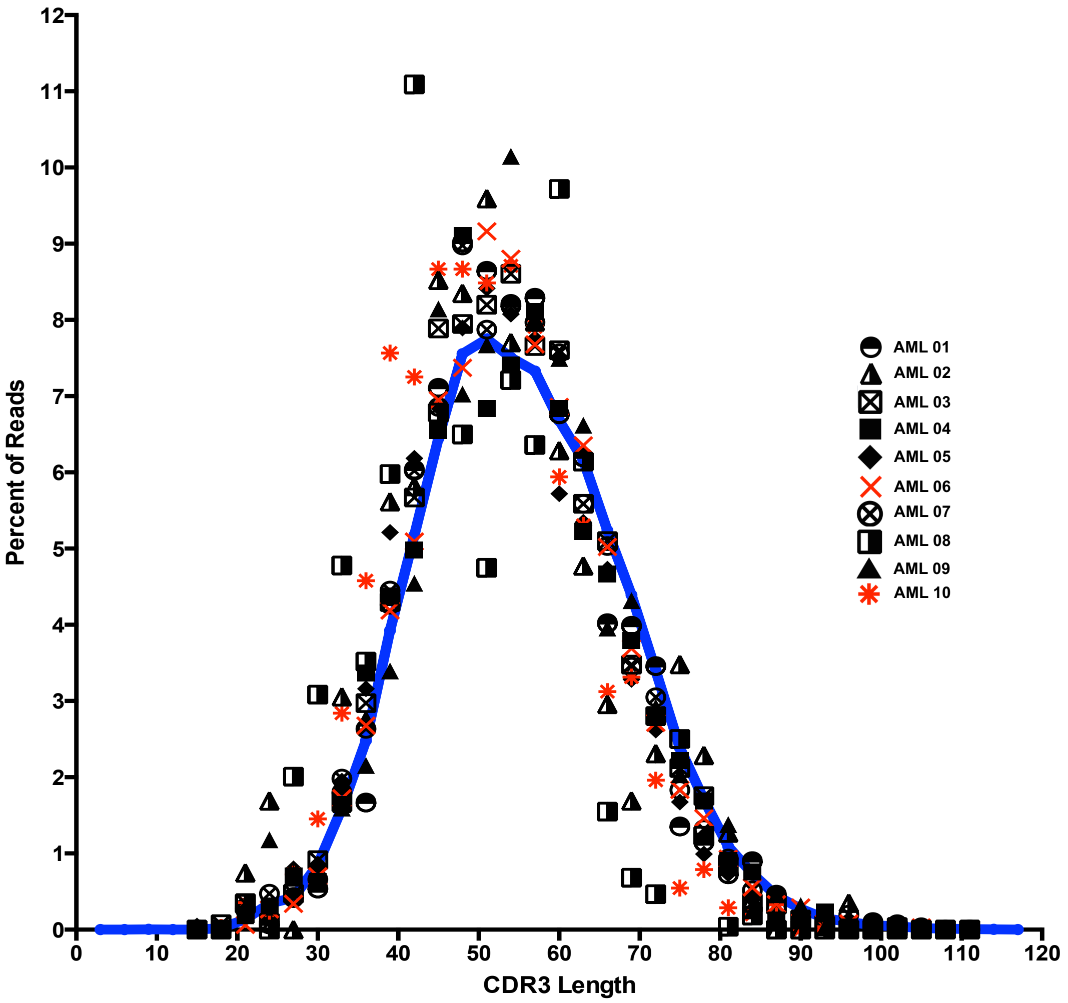

Fig. S6. Distribution of CDR3 lengths in productive rearrangements in AML patients (indicated in key) and 10 healthy individuals (blue points).

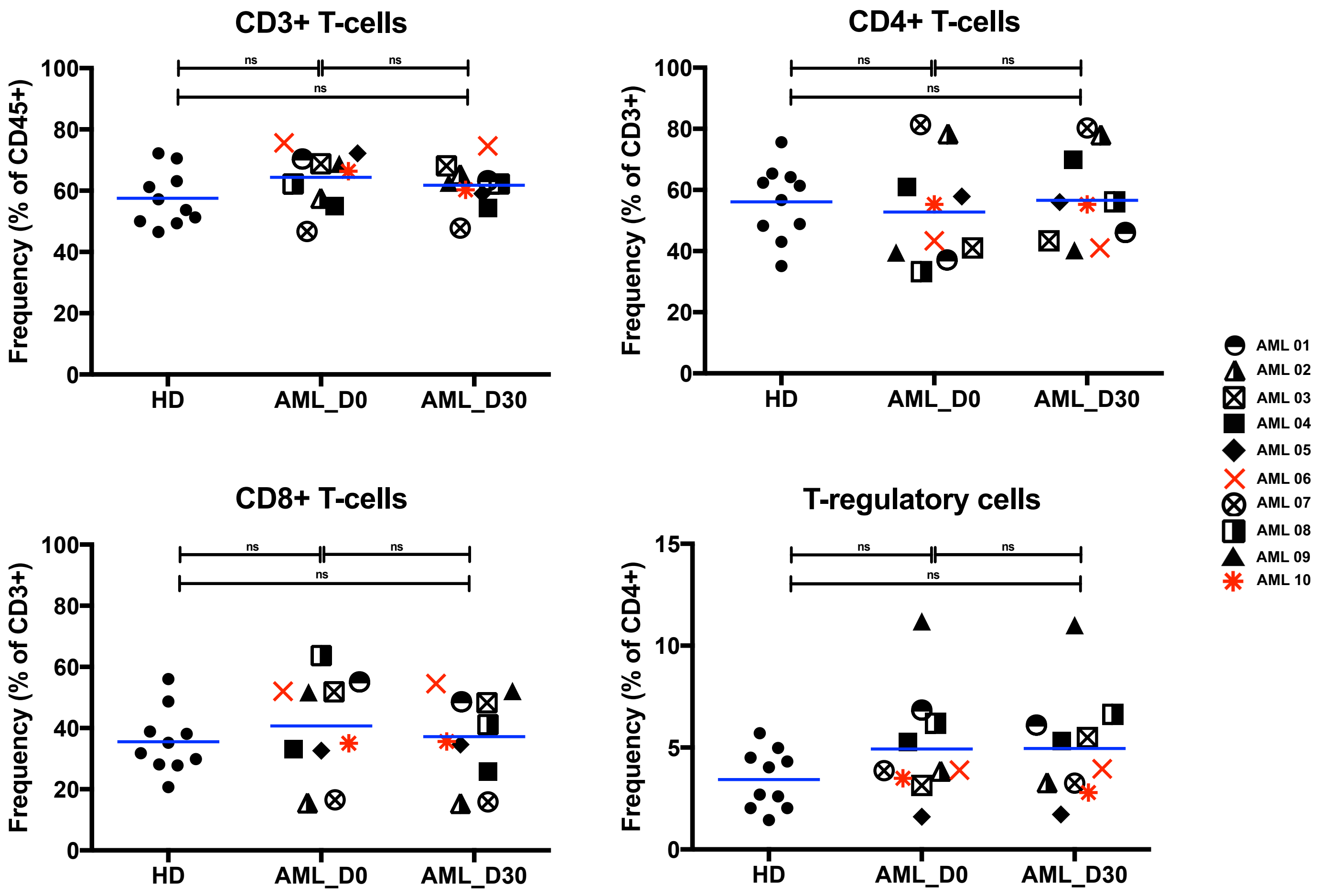

Fig. S7. Frequencies of T-cell populations in HD and AML at baseline and day 30. AML patients are indicated in the figure key. Blue lines indicate average frequencies for each group; “ns” indicates a non-significant difference.

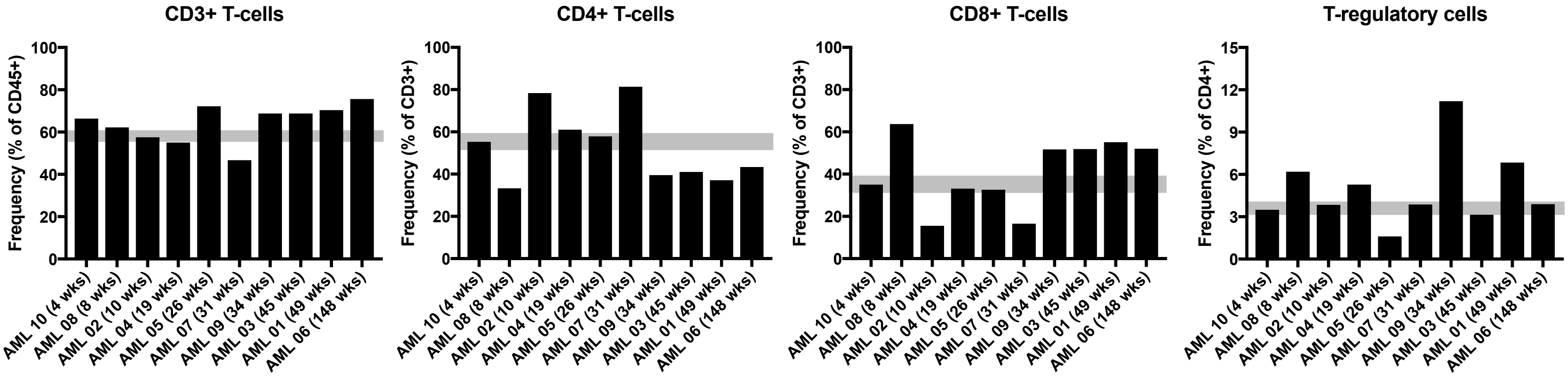

Fig. S8. AML patients at baseline are binned by time since treatment and indicated T-cell population frequencies plotted. Gray boxes highlight mean values +/- SEM of the HDs.

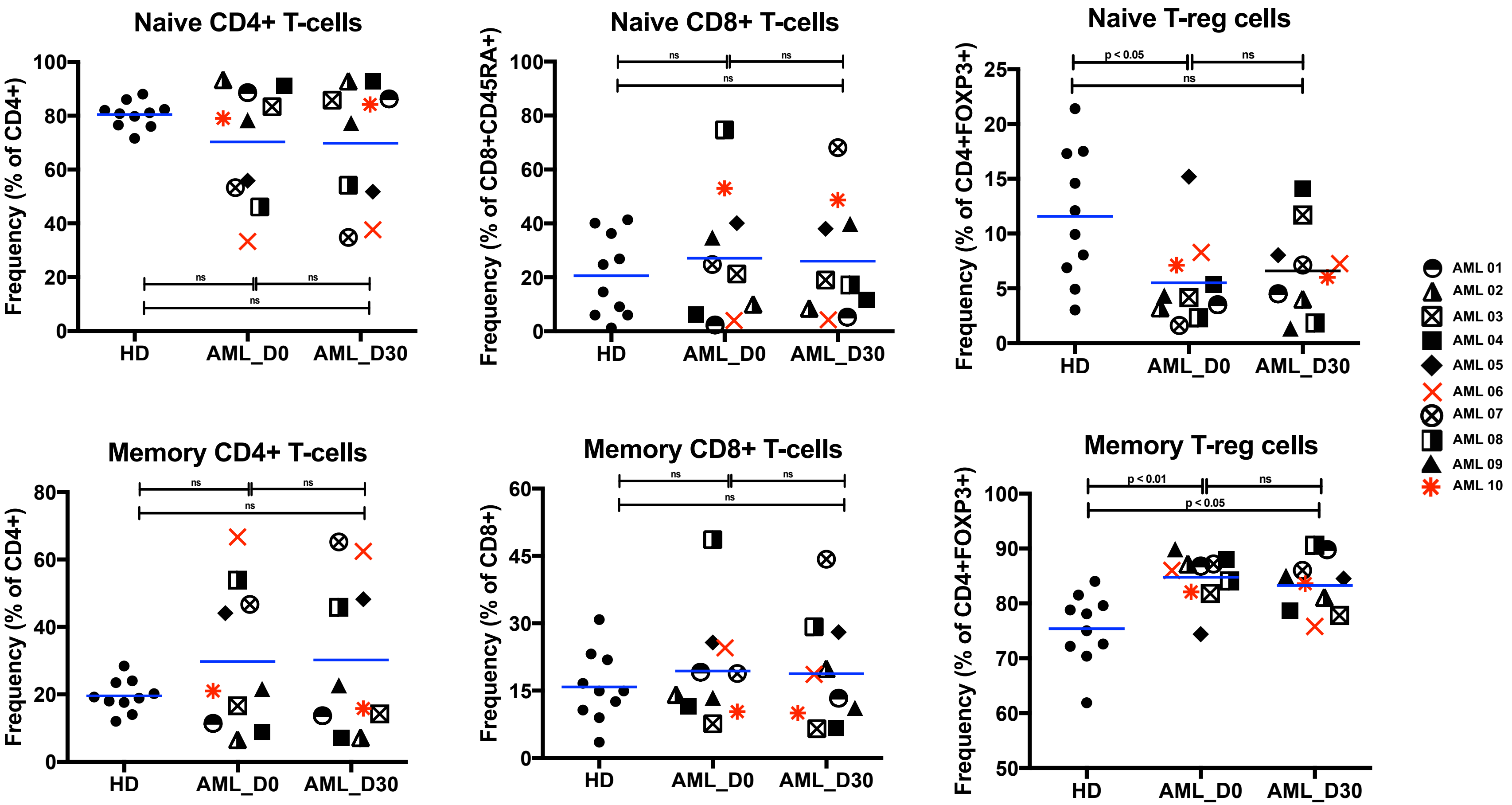

Fig. S9. Frequencies of indicated T-cells in HD and AML at baseline and day 30. AML patients are indicated in the figure key. Blue lines indicate average frequencies for each group; "ns" indicates a non-significant difference.

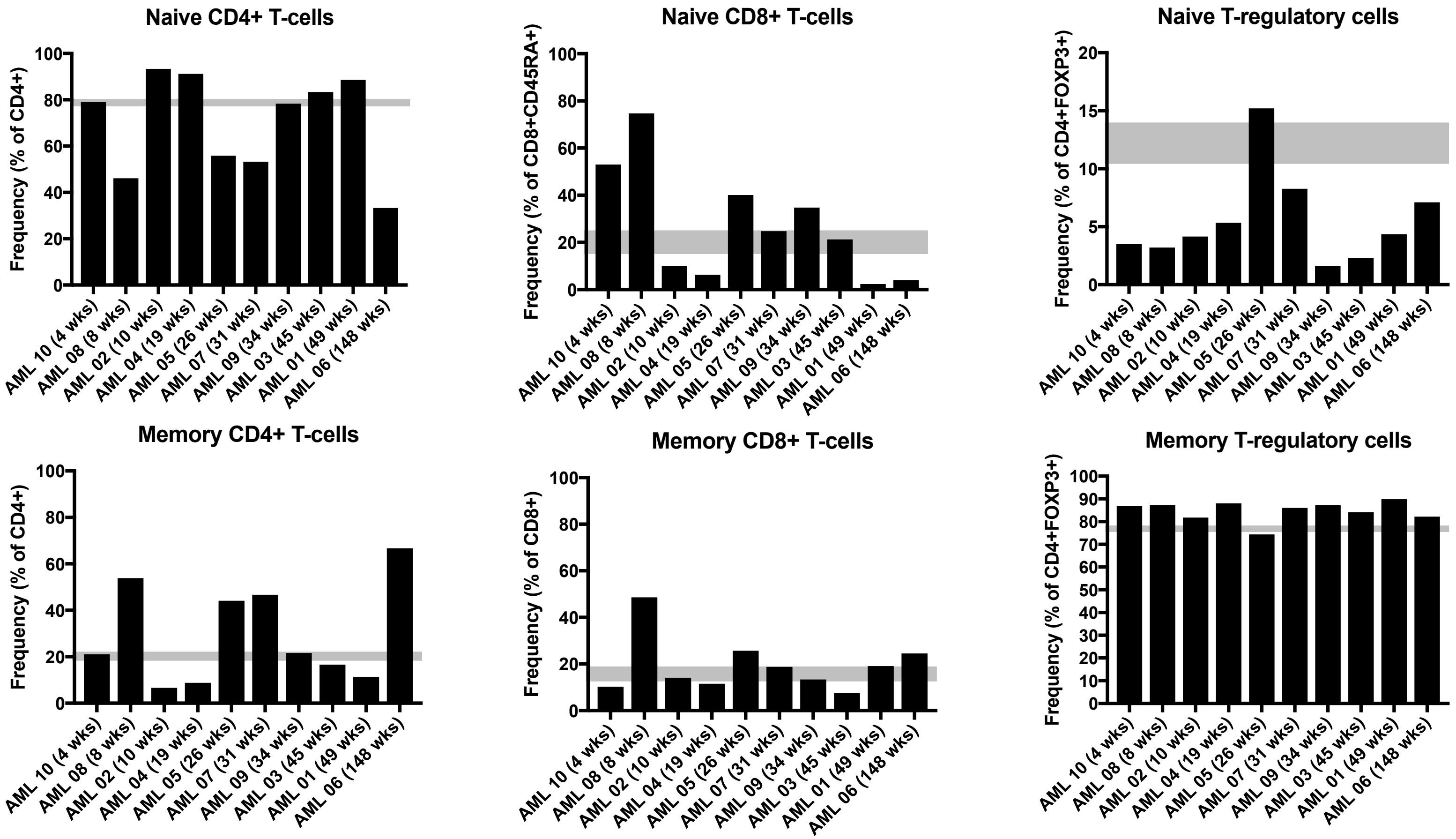

Fig. S10. AML patients at baseline are also ranked by time since end of chemotherapy and indicated T-cell population frequencies plotted. Gray boxes highlight mean values +/- SEM of the HDs.

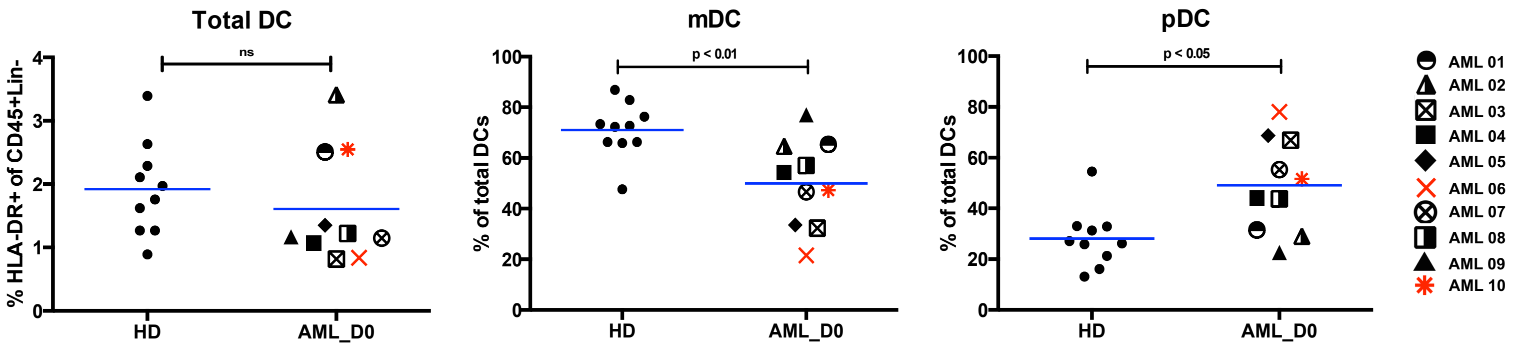

Fig. S11. Frequencies of total DCs, mDCs, and pDCs between HD and AML at baseline. AML patients are indicated in the figure key. Blue lines indicate average frequencies for each group; "ns" indicates a non-significant difference.
